# HIV-Associated Burkitt Lymphoma: Good Efficacy and Tolerance of Intensive Chemotherapy Including CODOX-M/IVAC with or without Rituximab in the HAART Era

**DOI:** 10.1155/2012/735392

**Published:** 2011-11-14

**Authors:** J. A. Rodrigo, L. K. Hicks, M. C. Cheung, K. W. Song, H. Ezzat, C. S. Leger, J. Boro, J. S. G. Montaner, M. Harris, H. A. Leitch

**Affiliations:** ^1^Division of Hematology, St. Paul's Hospital and the University of British Columbia, Vancouver, BC, Canada V6T 1Z4; ^2^Division of Hematology, St. Michael's Hospital and the University of Toronto, Toronto, ON, Canada M5S 1A1; ^3^Division of Hematology, Sunnybrook Health Sciences Centre and the University of Toronto, Toronto, ON, Canada M5S 1A1; ^4^Leukemia/BMT Program of British Columbia, BC Cancer Agency, Vancouver, BC, Canada V5Z 1M9; ^5^British Columbia Centre for Excellence in HIV/AIDS (CFE), St. Paul's Hospital and the University of British Columbia, Vancouver, BC, Canada V6T 1Z4; ^6^AIDS Research Program, St. Paul's Hospital and the University of British Columbia, Vancouver, BC, Canada V6T 1Z4

## Abstract

*Background*. The outcome of HIV-associated non-Hodgkin lymphoma (NHL) has improved substantially in the highly active antiretroviral therapy (HAART) era. However, HIV-Burkitt lymphoma (BL), which accounts for up to 20% of HIV-NHL, has poor outcome with standard chemotherapy. *Patients and Methods*. We retrospectively reviewed HIV-BL treated in the HAART era with the Magrath regimen (CODOX-M/IVAC±R) at four Canadian centres. *Results*. Fourteen patients with HIV-BL received at least one CODOX-M/IVAC±R treatment. Median age at BL diagnosis was 45.5 years, CD4 count 375 cells/mL and HIV viral load (VL) <50 copies/mL. Patients received PCP prophylaxis and G-CSF, 13 received HAART with chemotherapy and 10 rituximab. There were 63 episodes of toxicity, none fatal, including: bacterial infection, *n* = 20; grade 3-4 hematologic toxicity, *n* = 14; febrile neutropenia, *n* = 7; oral thrush; and ifosfamide neurological toxicity, *n* = 1 each. At a median followup of 11.7 months, 12 (86%) patients are alive and in remission. All 10 patients who received HAART, chemotherapy, and rituximab are alive. CD4 counts and HIV VL 6 months following BL therapy completion (*n* = 5 patients) were >250 cells/mL and undetectable, respectively, in 4. *Conclusion*. Intensive chemotherapy with CODOX-M/IVAC±R yielded acceptable toxicity and good survival rates in patients with HIV-associated Burkitt lymphoma receiving HAART.

## 1. Introduction

Burkitt lymphoma (BL) is a highly aggressive B-cell non-Hodgkin Lymphoma (NHL) associated with chromosomal translocations resulting in upregulation of the proto-oncogene C-MYC, which drives progression through the cell cycle [1]. It has an estimated incidence of 1200 patients per year in the United States [2]. Immunodeficiency associated BL is more commonly seen with human immunodeficiency virus (HIV) infection than other forms of immunodeficiency [3] though its incidence is lowest in patients with a CD4 count <50 cells/mL [4]. NHL accounts for approximately one third of AIDS-related malignancies and the frequency of BL is 2.4–20% of HIV-associated NHL [5].

 Several trials comparing the outcomes of patients with HIV-NHL have demonstrated improved outcomes in the HAART era [6–12]. Since the availability of rituximab (R), a monoclonal antibody directed against the B cell antigen CD20, outcomes have improved in HIV-negative B-cell lymphoma [13, 14]. In patients with BL or B-cell ALL treated with the intensive hyper-CVAD regimen; the addition of rituximab was identified in multivariate analysis as a favourable prognostic factor [15]. However, trials assessing the impact of rituximab in HIV-related NHL have shown mixed results [16–18]. An AIDS Malignancy Consortium (AMC) trial of CHOP versus CHOP-R for HIV-NHL showed a 14% rate of infectious deaths in the CHOP-R arm versus 2% with CHOP, offsetting an improvement in lymphoma control with CHOP-R [17]. However, in this study, HAART use was not uniform and most infectious deaths occurred in patients with a CD4 count <50 cells/mL. Conversely, a single institution review of patients treated with CHOP-like chemotherapy with or without rituximab for HIV-related diffuse large B-cell lymphoma (DLBCL) reported that CHOP-R was feasible in patients receiving HAART and yielded an overall survival (OS) of 86% at 30 months. This was superior to outcomes in patients receiving chemotherapy with HAART but no rituximab (*P* < 0.03), and the only toxic deaths seen with rituximab were in patients not receiving HAART [16]. A third study showed a 2-year OS rate of 75% in patients receiving rituximab with chemotherapy for HIV-associated NHL [19]. Finally, a recent trial from the AMC confirmed good tolerance of immunochemotherapy with or without HAART, though increased infectious deaths in patients with a CD4 count <50 cells/mL remained problematic [20].

In HIV-negative patients with BL, the most successful treatments are intensive multiagent chemotherapy protocols given over a short period to circumvent the development of drug resistance [19, 21–23]. Prior to the HAART era, HIV patients tolerated standard chemotherapy regimens poorly [24] and intensive chemotherapy was generally not feasible. However, a recent study of HIV-BL showed poor outcomes with standard chemotherapy, underscoring the need for intensification of therapy appropriate to the lymphoma [25]. Since the advent of HAART, intensification of chemotherapy in HIV-infected patients has been possible [26–28]. 

In 1996, Magrath et al. reported the use of the chemotherapy regimen CODOX-M/IVAC, which yielded a two-year event free survival of 85–92% in patients with BL [29]. In this study, we reviewed the outcomes of patients with HIV-associated BL who received intensive chemotherapy with the Magrath regimen and HAART, with or without rituximab. 

## 2. Patients and Methods

Patients treated with the Magrath regimen were identified from the database of the hematology practices [30]. Patients from two centers in Toronto, Ontario and two centers in Vancouver, British Columbia (BC) were included. All patients had biopsy proven BL and were HIV positive at lymphoma diagnosis.

Clinical characteristics and details of therapy were abstracted by chart review. Patient demographics, details of HIV infection and treatment, BL stage, toxicity of therapy, lymphoma response to therapy, and survival were recorded. Patients underwent standard diagnostic and staging investigations for lymphoma including: history and physical examination; excisional or core needle biopsy; blood counts and chemistry; lactate dehydrogenase (LDH) level; computed tomography of the chest, abdomen, and pelvis; bone marrow aspiration and biopsy. Data were collected as to site of lymphomatous involvement and largest mass, age, sex, Eastern Cooperative Oncology Group (ECOG) performance status (PS), initial and subsequent treatments, response to therapy, complications of therapy, and date and cause of death. Lymphoma diagnosis was determined by pathologists using standard diagnostic criteria [3] including identification of the t(8;14) and/or C-MYC translocation by karyotype analysis or fluorescence in situ hybridization (FISH). Pathology review was performed at the individual institutions at which the patients received treatment. BL risk was defined retrospectively by Magrath [29] and BC Cancer Agency criteria [30]. Low risk by Magrath criteria was ≤1 extranodal site of BL involvement; LDH level of ≤350 IU/L; all other patients were considered high risk. Low risk by BCCA criteria was Ann Arbor stage I, II, or III; bulk <5 cm; normal LDH level; all other patients were considered high risk. 

All patients were treated as high risk. The planned Magrath regimen involved two cycles of chemotherapy repeated for a total of four treatments (CODOX-M ± R (treatment A) followed by IVAC ± R (treatment B), then repeated). All patients received central nervous system (CNS) prophylaxis with intrathecal methotrexate (MTX) and cytarabine. One patient received EPOCH instead of the second IVAC-R treatment because of neurotoxicity from ifosfamide. Chemotherapy doses and schedules were CODOX-M ± R: cyclophosphamide 800 mg/m^2^ intravenously (IV) on days 1 and 2, doxorubicin 50 mg/m^2^ IV on day 1, vincristine 1.4 mg/m^2^ IV on days 1 and 8, methotrexate (MTX) 6720 mg/m^2^ [29] or 3000 mg/m^2^ IV on day 10 (MTX 3000 mg/m^2^ was adopted in BC as per BC Cancer Agency guidelines in 2006, the rationale being that 3000 mg/m^2^ MTX consistently penetrates the blood-brain barrier but may have less toxicity than higher doses) followed by standard leucovorin rescue [30]. Rituximab 375 mg/m^2^, when given, was on day 8. 

IVAC-R: cytarabine 2000 mg/m^2^ IV every 12 hours on days 1 and 2, ifosfamide 1500 mg/m^2^ IV given with MESNA on days 1–5, etoposide 60 mg/m^2^ on days 1–5, rituximab 375 mg/m^2^, if patients received it, was given on day 4.

EPOCH: etoposide 50 mg/m^2^ continuous IV infusion days 1–4, doxorubicin 10 mg/m^2^ continuous IV infusion days 1–4, vincristine 0.4 mg/m^2^ continuous IV infusion days 1–4, cyclophosphamide 375 mg/m^2^ IV day 5, prednisone 60 mg/m^2^ orally days 1–5, rituximab 375 mg/m^2^ on day 8.

Rituximab was given according to provincial availability; this medication was available for all ten BC patients but not for the four patients treated in Ontario.

For the first patient treated in BC and all Ontario patients (five patients in total), CNS prophylaxis or treatment was planned according to the Magrath regimen for high risk patients. This consisted of two intrathecal injections of cytarabine 70 mg and one injection of MTX 12 mg with cycle A and one MTX 12 mg with cycle B [29]. For subsequent patients from BC (nine patients total), prophylaxis was planned according to BCCA guidelines, which consisted of one injection of cytarabine 50 mg with cycle A and two injections of MTX 12 mg with cycle B [30]; two additional injections of cytarabine are generally given when feasible for a total of eight intrathecal injections.

Adverse events were graded using the National Cancer Institute Common Toxicity Criteria. Late neutropenia was defined as an absolute neutrophil count less than 0.5 × 10^9^/L at 12 weeks or greater following completion of all chemotherapy. Complete remission (CR) was defined as the disappearance of all evidence of lymphoma maintained for at least 4 weeks following the completion of therapy. Partial remission (PR) was defined as at least a 50% reduction in the sum of the largest diameters of all measurable lesions at 4 weeks following completion of all therapy. Progression was defined as the regrowth of previously responding lesions or the appearance of disease at a new site. Overall survival (OS) was defined as the time from diagnosis to the time of death from any cause. Patients were censored at the last known date of contact. OS was determined by the Kaplan-Meier method using SPSS for windows, version 17.0 (SPSS, Chicago, Ill, USA).

## 3. HIV Characteristics

Clinical data collected were HIV risk (sexual, injection drug use (IDU), etc.), CD4 count and HIV viral load at lymphoma diagnosis, prior AIDS, coinfection with the hepatitis B and/or hepatitis C viruses, and HAART use. HAART was defined as two nucleoside/nucleotide analogues and at least one protease inhibitor or a nonnucleoside reverse transcriptase inhibitor [31].

This study was performed in accordance with the requirements of the Institutional Research Ethics Board at each centre.

## 4. Results

Fourteen patients with HIV-associated BL diagnosed between December 2004 and August 2009 who received at least one treatment from the Magrath protocol were identified [30]. Patients were from St. Paul's Hospital, *n* = 7; Vancouver General Hospital, *n* = 3 (Vancouver); St. Michael's Hospital, *n* = 2; Sunnybrook Hospital, *n* = 2; (Toronto). One patient had features intermediate between DLBCL and BL according to the 2008 World Health Organization (WHO) classification, including a documented t(8;14) and a proliferation rate of 80% in the pericardial fluid. The clinical features were considered to be more in keeping with BL than DLBCL and he was treated as such. The C-MYC translocation was confirmed by FISH for MYC in nine patients, t(8;14) in eight, and FISH was unsuccessful in one. The t(8;14) was confirmed by karyotype analysis in one patient. The t(14;18) or BCL-2 was negative by: FISH for t(14;18) in four patients; FISH for BCL-2 in six patients and immunohistochemistry (IHC) for BCL-2 in three patients (one of these patients had focal weak positivity for BCL-2 by IHC, which is accepted in the WHO 2008 classification) [3]. The t(14;18) was negative by karyotype analysis in two patients, and BCL-2 and t(14;18) were not reported in two patients. 

The baseline characteristics of the patients are shown in Table 1. Median age at BL diagnosis was 45.5 (range 32–56) years and all patients were male. By Magrath risk criteria [29], eleven patients had high risk BL and three were low risk. By BCCA risk criteria [30], all fourteen patients had high risk BL. One patient had CNS involvement at BL diagnosis. One patient receiving the Magrath regimen had an ECOG performance status of 4 prior to chemotherapy; he improved dramatically with cyclophosphamide administered as a single agent and the remainder of the Magrath regimen was given starting on day 7 with a 50% dose reduction of doxorubicin for an increased bilirubin level as per BCCA guidelines [30].

Two patients with BL not receiving the Magrath regimen as initial therapy over the same time period were identified. The first had CNS involvement at BL diagnosis, was obtunded, had several comorbidities, and received palliation. The second was initially diagnosed as DLBCL and received EPOCH-R as initial therapy; the diagnosis was later amended to BL. The lymphoma progressed following cycle 4 of EPOCH-R, and he was switched to the Magrath regimen with initial control of BL. However, the lymphoma progressed within one month of completing the Magrath regimen, and he received palliative therapy thereafter.

The median CD4 count at BL diagnosis was 375 (range 140–760) cells/mL and HIV viral load <50 (<50–200 000) copies/mL. Ten patients were receiving HAART at BL diagnosis and 13 received HAART concurrent with chemotherapy. Ten patients received rituximab with chemotherapy and HAART. Seven patients received all four planned treatments with rituximab, one patient received three treatments (he declined further therapy and remains in remission at last followup), and two patients received only two rituximab treatments (one patient is still on treatment and in one patient the reason was not clear, but it was apparently not due to toxicity). Five patients received high dose MTX at 6720 mg/m^2^ as per the original Magrath protocol [29], and nine received 3000 mg/m^2^ as per BC Cancer Agency guidelines [30]. Thirteen patients received hematopoietic growth factor support between chemotherapy cycles with G-CSF. 

Six patients did not complete the entire four treatments of the Magrath regimen: two patients received three treatments (cycles 1A, 1B, and 2A) and four patients received two treatments (cycles 1A and 1B). Of two patients receiving three Magrath treatments, one received EPOCH-R instead of IVAC-R as the fourth treatment because of prior ifosfamide neurological toxicity. The other did not receive the second IVAC-R because of concern that there might be difficulty with collection of autologous stem cells for transplantation. This patient did not go on to transplant and declined further treatment, but did achieve complete remission and remains in remission at 27.6 months of followup. Two patients who received two Magrath treatments received additional cycles of lower intensity chemotherapy; one had a complete remission and one had no response. A third patient presented with CNS involvement and died of progressive BL following two Magrath treatments. He received eleven intrathecals in total in Magrath doses (cytarabine 70 mg and MTX 12 mg). The CSF was positive on only the first specimen in this patient, and all subsequent samples were negative for BL. Cycle 1B in this patient was complicated by anoxic brain injury secondary to sepsis, and he received palliation thereafter. The lymphoma appeared to be responding to treatment at the time that active therapy was discontinued. The fourth patient was still on treatment at the time of data analysis. 

Intrathecal prophylaxis or treatment received was as follows. One patient with positive CSF for BL received eleven intrathecal injections of chemotherapy (IT) in Magrath doses (cytarabine 70 mg, MTX 12 mg) though the number of each cytarabine and MTX doses given are uncertain. One patient received nine IT, with five cytarabine 50 mg and four MTX 12 mg. Three patients received eight IT, two patients with four cytarabine 70 mg and four MTX 12 mg, and one received four each of cytarabine 50 mg and MTX 12 mg. One patient received seven IT, with two cytarabine 50 mg and five MTX 12 mg. One patient received five IT, two cytarabine 50 mg, and three MTX 12 mg. Three patients received four IT; one patient received two cytarabine 50 mg combined with MTX 12 mg and four MTX 12 mg and two patients each received two cytarabine 50 mg and MTX 12 mg. Two patients received three IT. One received cytarabine 70 mg and MTX 12 mg, with the number of each uncertain. One received cytarabine 25 mg combined with MTX 9 mg (dose reduced for increased bilirubin level as per BCCA guidelines) then two MTX 12 mg; this patient declined further IT treatments. Finally, two patients received two IT. Both received two cytarabine 70 mg; one declined further IT treatments and one died of lymphoma.

Thirteen patients were documented to have received prophylaxis for PCP infection (trimethoprim-sulfamethoxazole, *n* = 10; dapsone, *n* = 2; not specified, *n* = 1), eight for herpes simplex virus/varicella zoster virus (HSV/VZV (valacyclovir, *n* = 6; acyclovir, *n* = 2)), and four for fungal infections (fluconazole, *n* = 2; amphotericin B, *n* = 2). Of three patients known to be hepatitis B positive, all received prophylaxis (emtricitabine, *n* = 2; lamivudine, *n* = 1).

The toxicity of treatment is shown in Table 2. No fatal toxic events were observed. The most common grade 3-4 adverse events were bacterial infection, *n* = 15; hematologic toxicity, *n* = 14; febrile neutropenia, *n* = 7. There were only two opportunistic infections, oral thrush and presumed HSV esophagitis.

One patient developed an altered level of consciousness after receiving two doses of ifosfamide. Mental status returned to normal within 48 hours after ifosfamide was held, but deteriorated when rechallenged and recovered fully within 36 hours with administration of methylene blue [32, 33]. This patient received the EPOCH-R regimen in substitution for cycle 2B. Two patients had elevated liver enzymes: one with known hepatitis C coinfection and the other had involvement of the biliary tract with BL at presentation. Dose reductions or changes in regimen were required in three patients because of therapy-related toxicity. Dose reductions and delays were as follows. Vincristine was held in cycle 2 due to severe peripheral neuropathy in one patient. One patient did not receive day 2 cyclophosphamide in cycle 1A due to developing a cardiac syndrome. In this patient, cycle 2A was given without incident. This same patient developed ifosfamide neurotoxicity with cycle 1B. He received 2 of 5 doses of ifosfamide, 3 of 5 doses of cytarabine, and completed cycle 1B with day 3–5 etoposide given on days 15–17. For cycle 2B he received EPOCH-R. One patient presented with a bilirubin level of 361 (normal < 20) umol/L from BL hepatic infiltration. He received dexamethasone 4 mg four times daily (from day-1) followed by cyclophosphamide 1000 mg/m^2^ (day 1) and by day 6 the bilirubin was 67. He received the remainder of day 1-2 chemotherapy on day 7 (doxorubicin was given at a 50% dose reduction for increased bilirubin as per BCCA guidelines), rituximab on day 8, and high dose MTX on day 15. The bilirubin normalized by day 26. One patient who received full Magrath doses of MTX had cycle 2A high dose MTX delayed by 6 days because of grade 4 neutropenia. Late neutropenia occurred in five patients; all responded to administration of G-CSF.

Toxicity did not appear to occur more frequently according to the type of HAART used, for example comparing protease-inhibitor (PI) based to non-PI-based regimens, the occurrence of any toxicity, the number of toxic episodes, peripheral neuropathy, increased liver function tests, and late neutropenia did not differ between groups, nor did the requirement for chemotherapy dose reductions. The episode of oral thrush, mucositis, and skin reaction (one each), however, all occurred in patients receiving PI-based HAART.

CD4 counts and HIV VL measurement were available six months following the completion of chemotherapy in five patients; the CD4 count was >250 cells/mL and HIV VL <50 copies/mL in four.

## 5. Survival and Causes of Death

**Figure 1:**
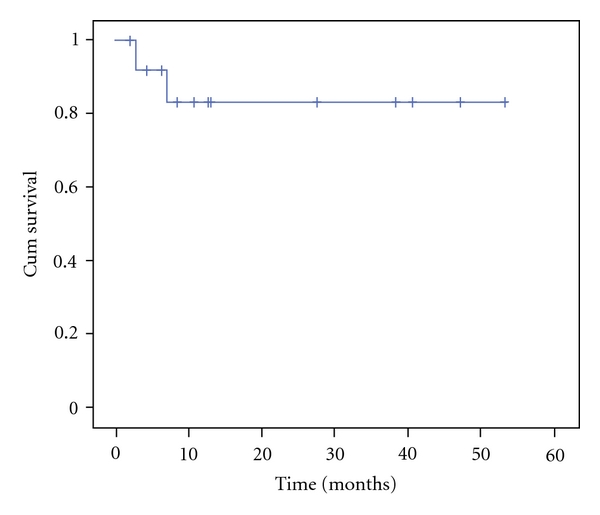
Overall survival of 14 patients with HIV-associated Burkitt lymphoma receiving CODOX-M/IVAC chemotherapy, 13 with HAART and 10 with rituximab.

At a median followup of 11.7 (2.0–53.2) months, 12 of 14 patients (86%) are alive and in remission (Figure 1). All 10 patients who received HAART, intensive chemotherapy, and rituximab are alive. Eleven of 12 survivors had high-risk BL and 10 had a CD4 count >200 cells/mL at BL diagnosis. There were 2 deaths, at 2.9 and 6.9 months from lymphoma diagnosis, both from progressive lymphoma. The CD4 count at BL diagnosis in these patients was 140 and 180 cells/mL. One patient presented with CNS involvement by BL and received chemotherapy without HAART or rituximab. The second patient received chemotherapy and HAART but no rituximab, and received only two cycles of therapy as he suffered anoxic brain injury secondary to sepsis, prompting a change in direction of care to palliative management. He ultimately died of progressive BL. 

## 6. Discussion

In recent years, there has been a shift in treatment goals for patients with HIV-associated NHL. Prior to the HAART era, infectious deaths occurred frequently, as immunosuppression and myelosuppression from HIV made intensive chemotherapy regimens needed to effectively treat aggressive lymphomas difficult to deliver and lower-dose chemotherapy regimens given with palliative intent were recommended [24]. In the HAART era, it has become clear that standard dose chemotherapy for DLBCL [16, 34], and now intensive regimens for BL can be considered and used with success [26, 28, 35–37]. In this study, we reviewed 14 HIV-positive patients with BL treated with CODOX-M/IVAC±R. Most of our patients were receiving HAART at BL diagnosis and this was reflected in their relatively preserved immune parameters; the median CD4 count was 375 cells/mL and HIV VL <50 copies/mL. Previous series [38] have shown some success with intensive chemotherapy in HIV-associated BL. Wang et al. compared patients infected with HIV (*n* = 8 patients) to HIV-negative patients treated with the same regimen and found that toxicity from CODOX-M/IVAC was similar between groups, with similar rates of myelosuppression and infectious complications to HIV-negative patients [38]. A recent study of 30 patient receiving CODOX-M/IVAC and HAART showed a 3-year OS rate of 52% [39]. In addition, in a recent update from the AMC, 33 patients with HIV-associated BL were treated with modified CODOX-M/IVAC-R in which high dose MTX was given at 3000 mg/m^2^. At a median followup of 9 months, the one year OS was 82% with no treatment related mortality [40]. Similarly, in 29 BL patients treated with dose-adjusted EPOCH-R, 10 of whom were HIV-positive, at a median followup of 57 months, the OS was 100% [41]. Our CR rate of 86% compares favorably with this experience, as does the projected one and two year OS of 83% (median followup 11.7 months) and tolerability of the regimen. The outcomes are similar to those described in HIV-negative patients with BL [42]. Relapses of BL tend to occur early, within a few months of diagnosis [43]. At a median followup of 11.7 months, only two patients died, both within seven months of diagnosis, and both of BL. Of the twelve other patients, none have relapsed, though two are less than six months from BL diagnosis. Moreover, ten of twelve survivors had high-risk features at presentation by Magrath criteria and all twelve survivors were high risk by BCCA criteria, suggesting that this therapeutic approach can overcome high-risk BL. 

The role of immunotherapy with rituximab added to chemotherapy in HIV-BL has been a topic of discussion, though updated results from the AMC trial indicate that this agent can be safely administered concurrently with chemotherapy (EPOCH) and HAART, with good outcomes [20]. Our patients received at most four doses of this agent. The combination of eight doses of rituximab with the hyper-CVAD regimen in HIV-negative patients with BL was compared to historical patients treated with hyper-CVAD alone. There was a significant reduction in relapse rate favoring the inclusion of rituximab (7% versus 34%, *P* = 0.008), and improved 3-year OS (89% versus 53%, *P* < 0.01) [15]. Eight doses of rituximab have also been combined with an intensive chemotherapy regimen in a cohort of HIV-positive and HIV-negative patients with similar results, including a CR of 88% and 84%, respectively [36]. Whether more than four doses of rituximab included with short-course high-intensity chemotherapy such as CODOX-M/IVAC would confer additional benefit is unknown but may be an area worthy of future investigation.

In our ten patients receiving rituximab with BL therapy, the only evidence of additional complications was the occurrence of late neutropenia in five; all responded to G-CSF. This complication appears to be rituximab related, as has been described in HIV-negative patients. Although late neutropenia appeared to be equally distributed among patients receiving PI-based versus non-PI-based HAART, interactions between antiretroviral agents and chemotherapy medications resulting in increased marrow toxicity cannot be ruled out [44, 45].

Another toxicity noted in HIV-DLBCL with rituximab was an increase in herpes virus infections [16]. For HIV-positive patients receiving rituximab with chemotherapy for NHL, we recommend HSV/VZV prophylaxis and monitoring for cytomegalovirus reactivation in those with culture negative fever. Although these measures were documented in only eight of our BL patients, there was only one presumed herpes virus infection in the current study. In the AMC trial, a higher incidence of infectious toxicity was associated with rituximab in patients with a CD4 count of <50 cells/mL [17]. Although none of our patients had a CD4 count <50 cells/mL at BL diagnosis, all ten patients who received rituximab with intensive chemotherapy and HAART are alive. It should be noted that rituximab in patients with active Kaposi sarcoma (KS) may result in severe KS flares [46]. 

In general, the toxicity of therapy experienced by our patients, largely bacterial infections, febrile neutropenia and grade 3-4 bone marrow suppression, was in keeping with what one would expect in HIV-negative patients receiving this chemotherapy protocol. Opportunistic infections occurred in only two patients, oral thrush in one patient and presumed HSV esophagitis in another. The only HIV-specific form of prophylaxis routinely given was for PCP, which was given regardless of CD4 count, since the CD4 count may decrease on chemotherapy. However, Montoto et al. documented a CD4 count >200 cell/mL and undetectable HIV VL at six months following the completion of BL therapy in 58% and 88% of patients, respectively, indicating good immunological recovery and virological control despite intensive chemotherapy, and our findings are in keeping with this [39].

There was a low rate of mucositis in this series, with two patients experiencing grade 1-2 stomatitis, and a third patient experiencing stomatitis with the grade not specified. One patient was treated for presumed grade 3 HSV esophagitis, and it is possible that this patient actually had mucositis. Of note, nine of the 14 patients in this series received high-dose MTX at 3000 mg/m^2^ [30] as compared to the dose of 6720 mg/m^2^ used in the original Magrath protocol [29], and this could have resulted in lower than expected rates of mucositis. However, the possibility that episodes of grade 3-4 mucositis were not clearly documented and recognized retrospectively cannot be ruled out.

 More than half of patients (8 of 14) received all four planned Magrath treatments. Of six who received fewer intensive treatments, only two were due to toxicity. One patient had ifosfamide neurotoxicity from which he fully recovered [32, 33]; he received EPOCH-R as cycle 2B and had no evidence of lymphoma at four months. A second patient suffered anoxic brain injury secondary to sepsis and was subsequently palliated. These results are in contrast to our experience with HIV-BL in the pre-HAART era; although not fully documented and formally compared, many of our patients suffered toxic deaths despite suboptimal lymphoma chemotherapy.

Limitations of this study include its retrospective nature and small number of patients reviewed. As the study was nonrandomized, selection bias must be considered, as these results may not apply to all patients with HIV-BL. The 14 patients reported here, however, were all the HIV-BL seen over this time period with two exceptions. The first had CNS involvement at BL diagnosis, a very poor performance status, and received palliation. The second had an initial diagnosis of DLBCL made which was later amended to BL; because of the initial diagnosis, the Magrath regimen was not used as first-line treatment and this patient was not included for purposes of this report. Thus, the patients reported here represent a reasonably unselected group of HIV-BL seen over this time period. Even though two patients with HIV-BL during this period did not receive the Magrath regimen, some who did receive Magrath did not receive all planned cycles or rituximab and one did not receive HAART, CODOX-M/IVAC chemotherapy with HAART and rituximab was feasible in the majority of patients, was well tolerated by most, and resulted in acceptable lymphoma control and reasonable immunological recovery and virological suppression. As with other NHL in the HAART era [47], our data suggest that the clinical outcome of BL has improved to the point that it may be comparable to outcomes in HIV-negative patients with similar lymphomas [36, 37].

## 7. Conclusion

In this review of patients with HIV-associated BL treated with the intensive Magrath (CODOX-M/IVAC) chemotherapy regimen and HAART, patients had acceptable tolerance of therapy even when it included rituximab. Of ten patients who received chemotherapy, rituximab, and HAART, none has died. Eleven of twelve survivors had high-risk features, suggesting that this therapeutic approach can overcome high-risk BL. These results suggest that if HIV control is optimized, patients with HIV-associated BL who receive intensive chemotherapy and rituximab could achieve survival similar to HIV-negative BL patients without undue therapy-related toxicity. 

## Figures and Tables

**Table 1:** Clinical characteristics and initial treatment of 14 patients with HIV-associated Burkitt lymphoma.

Characteristic	*n*
Age at BL presentation (years)	
≤45	7
>45	7
Age in years, median (range)	
45.5 (32–56)	
Gender	
Male	14
BL stage	
I	2
II	1
III	4
IV	7
Magrath risk^1^	
Low	3
High	11
BCCA risk^2^	
Low	0
High	14
LDH	
Normal	6
Increased	8
ECOG PS^3^	
0-1	4
≥2	5
Extranodal sites	
≥1	7
HIV risk^4^	
Sexual	9
IDU	3
CD4 at BL diagnosis	
<200	4
≥200	10
Prior AIDS^5^	
No	13
Yes	1
Coinfections	
Hepatitis B	
Known negative	9
Known positive	3
Hepatitis C	
Known negative	10
Known positive	3
HAART^6^	
No	1
Yes	13
G-CSF	
Yes	13
Number of cycles of HD-CT	
1-2	4
3-4	10
Received rituximab	
No	4
Yes	10

^1^Magrath risk: low risk has ≤1 extranodal site of BL and LDH ≤350 IU/L; all others are high risk.

²BCCA risk: low risk has Ann Arbor stage I, II, or III; bulk <5 cm; normal LDH level; all others are high risk.

^3^ECOG Performance Status, *n* = 5 not recorded.

^4^HIV Risk, *n* = 2 not recorded.

^5^Kaposi sarcoma, *n* = 1.

^6^HAART usually includes one nucleoside analog, one protease inhibitor, and either a second nucleoside analog or a nonnucleoside reverse transcription inhibitor (NNRTI).

AIDS: acquired immunodeficiency syndrome; BCCA: British Columbia Cancer Agency; BL: Burkitt lymphoma; ECOG PS: Eastern Cooperative Oncology Group; G-CSF: granulocyte colony stimulating factor; HIV: Human Immunodeficiency virus; HAART: highly active antiretroviral therapy; HD-CT: high dose chemotherapy; IDU: injection drug use; LDH: lactate dehydrogenase; *n*: number of patients.

**Table 2:** Treatment-related toxicity in 14 patients with HIV-related Burkitt lymphoma receiving intensive chemotherapy with CODOX-M/IVAC ± rituximab.

Treatment-related toxicity	Grade 1-2		Grade 3-4	
	*n* (episodes)	*n* (patients)	*n* (episodes)	*n* (patients)
Bacterial infection^1^	5	3	15	4
Culture negative febrile neutropenia	—	—	7	7
Late neutropenia	5	4		
Opportunistic infection^2^	1	1	1	1
Grade 3 or 4 hematotoxicity	—	—	14	14
Cardiac syndrome^3^	—	—	1	1
Stomatitis	3	3^4^	—	—
Increased liver enzymes	2	2	—	—
Skin reaction	1	1	—	—
Peripheral neuropathy	1	1	1	1
Hallucinations	1	1	—	—
Neurotoxicity from ifosfamide	—	—	1	1
Chemotherapy dose reductions, delays, or changes due to toxicity^5^	—	—	5	4

^1^Included: bacteremia, *n* = 12 episodes in 7 patients; urinary tract infection, *n* = 4 in 2 patients; clostridium difficile diarrhea; *n* = 3 in 3 patients; cellulitis, *n* = 1 in 1patient.

^2^Oral thrush in 1 patient, presumed HSV esophagitis in 1 patient.

^3^Poorly defined cardiac syndrome; possible CHF following day 1 of cycle 1A, requiring admission to the Coronary Care Unit; patient recovered and completed treatment modified for other toxicities.

^4^In one of these patients the grade was not reported.

^5^Dose reductions/delays: Vincristine was held in cycle 2 due to severe peripheral neuropathy in one patient. One patient did not receive day 2 cyclophosphamide in cycle 1A due to developing a cardiac syndrome, in this patient cycle 2A was given without incident. One patient presented with a bilirubin level of 361 (normal < 20) umol/L from BL hepatic infiltration. He received dexamethasone 4 mg qid (day-1) followed by cyclophosphamide 1000 mg/m^2^ (day1), and by day 6 the bilirubin was 67. He received the remainder of day 1-2 chemotherapy on day 7 (doxorubicin was given at 50% dose for increased bilirubin as per BCCA guidelines), rituximab on day 8 and high dose MTX on day 15. The bilirubin normalized by day 26. The patient who had a cardiac syndrome with cycle 1A later developed ifosfamide neurotoxicity with cycle 1B. He received 2 of 5 doses of ifosfamide, 3 of 5 doses of cytarabine, and completed cycle 1B with day 3–5 etoposide given on days 15–17. For cycle 2B, he received EPOCH-R. One patient receiving full Magrath doses had cycle 2A high dose MTX delayed by 6 days because of grade 4 neutropenia.
